# Infrared Nanospectroscopy Reveals DNA Structural Modifications upon Immobilization onto Clay Nanotubes

**DOI:** 10.3390/nano11051103

**Published:** 2021-04-24

**Authors:** Federica Piccirilli, Franco Tardani, Annalisa D’Arco, Giovanni Birarda, Lisa Vaccari, Simona Sennato, Stefano Casciardi, Stefano Lupi

**Affiliations:** 1 Istituto Officina dei Materiali CNR, 34149 Trieste, Italy; stefano.lupi@roma1.infn.it; 2 Istituto dei Sistemi Complessi (ISC)-CNR, UOS Roma Sapienza, 00185 Roma, Italy; franco.tardani@gmail.com (F.T.); simona.sennato@roma1.infn.it (S.S.); 3 Dipartimento di Fisica, “La Sapienza” Universitá di Roma, 00185 Roma, Italy; annalisa.darco@roma1.infn.it; 4 National Institute of Nuclear Physics Section Rome, P.le A. Moro 2, 00185 Roma, Italy; 5 Elettra Sincrotrone Trieste, 34149 Trieste, Italy; giovanni.birarda@elettra.eu (G.B.); lisa.vaccari@elettra.eu (L.V.); 6 Dipartimento di Medicina, Epidemiologia, Igiene del Lavoro e Ambientale, Istituto Nazionale per l’Assicurazione Contro gli Infortuni sul Lavoro, 00100 Roma, Italy; s.casciardi@inail.it

**Keywords:** nanotubes, nano-FTIR, DNA

## Abstract

The growing demand for innovative means in biomedical, therapeutic and diagnostic sciences has led to the development of nanomedicine. In this context, naturally occurring tubular nanostructures composed of rolled sheets of alumino-silicates, known as halloysite nanotubes, have found wide application. Halloysite nanotubes indeed have surface properties that favor the selective loading of biomolecules. Here, we present the first, to our knowledge, structural study of DNA-decorated halloysite nanotubes, carried out with nanometric spatially-resolved infrared spectroscopy. Single nanotube absorption measurements indicate a partial covering of halloysite by DNA molecules, which show significant structural modifications taking place upon loading. The present study highlights the constraints for the use of nanostructured clays as DNA carriers and demonstrates the power of super-resolved infrared spectroscopy as an effective and versatile tool for the evaluation of immobilization processes in the context of drug delivery and gene transfer.

## 1. Introduction

Spindle-like nanostructures (such as carbon or clay nanotubes) are promising candidates as gene nanocarriers due to a remarkable penetration capability. A needle-like mechanism [1] or Yoshida effect [2] in the presence of shear forces allows the nanotubes to cross biological barriers without endosomal entrapment. Naturally-occurring clay nanotubes, such as halloysite nanotubes (HNs), offer in addition the advantage of abundance, low cost and high biocompatibility [3,4,5].

Previous investigations reported on the loading of different types of nucleic acids derivatives onto the outer surface on HNs [6,7,8,9]. The first immobilization was obtained by simple mechanic-chemical mixing of DNA with pristine HNs [6]. According to the authors, DNA is bonded through weak interactions without substantial modification. However, since HNs’ outer surface is negatively-charged, spontaneous DNA adsorption has been considered unexpected [10]. More recently, the immobilization of nucleic acid derivatives has been obtained by electrostatic attractive interactions after HN surface grafting with positively-charged functional groups [7,9].

All the above examples propose the use of pristine or specifically surface-modified HNs for application in drug and gene delivery [11,12]. Since HNs have a natural origin, a large variation in the chemical and structural characteristics has been observed [13], and it can have an impact on the adsorption of nucleic acids. Due to the relevant influence that the amount and the structural characteristics of adsorbed nucleic acids have on gene delivery efficency, as observed in lipid-based nanocarriers and in recent investigations on HNs [14,15,16], detailed investigation on DNA interaction and immobilization on nanocarriers is mandatory. UV-absorption and thermal gravimetric analysis can be employed for detecting DNA immobilization on nanoclays [17,18]. However, both techniques show a huge indetermination in quantifying the amount of immobilized molecules [18]. In addition, no spatial information about the DNA adsorption site can be obtained [17]. Electron microscopies (EM) could provide information complementary to the ones given by the aforementioned techniques. Although EM can probe the spatial distribution of immobilized molecules on nanoclay surfaces, the very complicated data analysis and sample damaging due to the e-beam exposure make the techniques not optimal for the study of these systems [19]. Interestingly, non-destructive high-resolution atomic force microscopy coupled to force spectroscopy investigation has proven useful for revealing DNA molecules adsorbed onto nanotubes [20] and paved the way for taking advantage of combined AFM with nanoscale-resolved spectroscopic mode. The coupling of spectroscopies and microscopies represents the best approach to studying HNs nanocarriers, allowing one to probe the system from two distinct points of view: chemico-physical and morphological.

Among all spectroscopies, Fourier Transform Infrared Spectroscopy (FTIR) has proven to be particularly useful for the structural analysis of biomolecules [21,22,23], since it can provide important information about the physico-chemical state of the main functional groups of biological matter. Moreover, infrared spectroscopy was successfully exploited for studying the DNA structural modifications that may originate from interactions with clay materials [6]. Microspectroscopic studies can be performed thank to the coupling of FTIR spectrometers to IR microscopes. However, the diffraction limit imposed to IR microscopy restricts the lateral resolution to a few microns [24,25], hampering the detection of nanoscopic details and single molecules.

**Figure 1:**
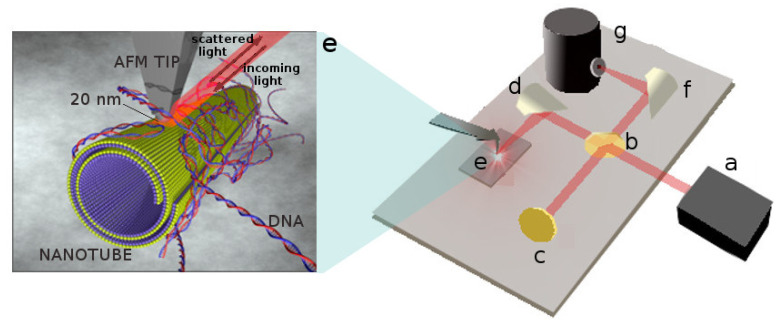
Schematic representation of the nano-FTIR apparatus: (**a**) DFG (Difference Frequency Generation) broadband laser source working in the mid-infrared range; (**b**) KBr beam splitter; (**c**) Moving mirror for Fourier transfom; (**d**) Focusing parabolic mirror aimed at focusing the incoming beam to the tip apex; (**e**) Nanoscopic conductive tip (PtIr) and DNA–HNs sample; (**f**) Parabolic mirror focusing light to the detector; (**g**) MCT (Mercury Cadium Tellurium) liquid nitrogen cooled detector for mid-infrared.

This limit is circumvented by nano-FTIR (Fourier transform infrared nanospectroscopy), an advanced technique based on scattering-type scanning near-field optical microscopy (s-SNOM) [26] that allows one to simultaneously obtain morphological and chemical information at the nanoscale. With nano-FTIR, infrared spectra with lateral resolution of few tens of nanometers are obtained by recording the infrared light scattered at a scanning probe tip (see Figure 1 for a schematic description of the technique).

The probe is typically a conductive atomic force microscope (AFM) tip that, acting as an optical antenna, squeezes the incident field at the tip apex [27]. When the tip is illuminated by the broadband infrared radiation, Fourier transform of the scattered light yields infrared spectra with a spatial resolution down to 20 nm [26,27,28].

Here, we report the first investigation of DNA-covered HNs by Fourier transform infrared nanospectroscopy. We directly observe that a certain amount of DNA is spontaneously immobilized on the HNs’ outer surface. This adsorption, however, produces some conformational modification of DNA. This information is of fundamental importance for the optimization of HN-based carriers and, more in general, for the study of nanocarriers systems for drug delivery and gene transfer. Given the unicity of the infrared fingerprint of molecules and the nanoscale spatial resolution of nano-IR, our results propose nano-IR spectroscopy as an excellent candidate for the characterization of loading and adsorption processes for a wide range of molecules and carriers.

## 2. Materials and Methods

Halloysite nanoclays and Calf Thymus DNA sodium salt were purchased from Sigma Aldrich (St Louis, MO, USA) and used without further purification. Morphological properties of the sample used in the present investigation have been accurately measured by Transmission Electron Microscopy. Results have been reported in Supplementary Information of our previous work [29]. We found that HNs have an average length of 400 nm, an average outer diameter of 50 nm and lumen size of 13 nm. Calf thymus-DNA is a linear chromosomal DNA with $10^{4}$ base pairs as determined by gel electrophoresis [30].

### 2.1. HN Dispersion

HNs were dispersed in MilliQ water by a sonication procedure. A volume of 10.0 mL of MilliQ water was added to 5.0 mg of HNs powder. The dispersion was initially vortexed for 30 s and subsequently tip-sonicated for 20 min. In order to avoid excessive heating, sonication was performed in an ice bath by following a two-step procedure (10 + 10 min). A pulse mode, with an amplitude of 20% (corresponding to a 4 W power) and with 1 s-1 s ON-OFF is used for sonication. The procedure, coupled to the two-step sonication, was chosen to allow maximum degree of debundling and, at the same time, to avoid damage of the nanotubes (as checked by TEM microscopy), as described in our previous investigation [29].

### 2.2. HNs–DNA Mixing

Dispersion of DNA-covered HNs was obtained by mixing a DNA solution (0.8 mg/mL) with an HNs dispersion (0.05 wt%) in a volume ratio of 1:9. The resulting solution was left equilibrating overnight in a vortex mixer at a speed set to 4 rpm. The final dispersion was subsequently added with 100 µL per 1.0 mL of a 70 mM NaCl solution to favor sedimentation, in accordance with recently developed protocols [29,31]. The system was then centrifuged at 24,100× *g* for 1 h. Subsequently, the supernatant solution was discarded, while the pellet was redispersed in MilliQ water. The solution was tip-sonicated for 30 min. In order to avoid excessive heating, sonication was performed in an ice bath by following a three-step procedure (15 min per step). A pulse mode, with an amplitude of 20% (corresponding to a 4W power) and with 1–1 s ON–OFF was used for sonication. The procedure, coupled to the three-step sonication, was chosen to allow a maximum degree of debundling and, at the same time, to avoid damage to the nanotubes (as checked by TEM microscopy), as described in our previous investigation [29]. According to the protocol of [29], the calculated amount of adsorbed DNA was 200 mg/g.

### 2.3. ATR-FTIR Measurements

ATR-FTIR measurements were performed using a Vertex70 interferometer (Bruker Optics, Ettlingen, Germany) equipped with a thermal source (Globar), an ATR crystal (Bruker-Germanium Multiple reflection crystal), and a deuterated triglycine sulfate (DTGS) detector. HNs, DNA–HNs, and DNA solutions were prepared according to the protocols reported in Section 2.1 and Section 2.2. A total of 10 µL was then dropped and measured after complete drying under gentle nitrogen flux. Measurements were acquired with an acquisition rate of 5 kHz in the range between 400 and 4000 cm${}^{-1}$ by averaging 256 scans. Spectra were corrected for atmospheric absorption, and a baseline was subtracted by using a Rubberband correction algorithm. Second-derivative analysis was performed, applying a 9-points smoothing filter [32,33]. Data postprocessing was performed using OPUS PRO 7.5 software (Bruker).

### 2.4. Nano-FTIR Measurements

Nano-FTIR measurements were carried out at the SISSI beamline (Elettra Sincrotrone Trieste and IOM CNR, Basovizza (TS), Italy) through the help of a nano-FTIR spectroscope (Neaspec, Munich-Haar, Germany) coupled to a DFG laser source operating in the range between 1100 and 1750 cm${}^{-1}$ and having an average power of 1200 µW. The microscope allowed us to simultaneously perform nano-FTIR and AFM measurements. Metallic tips (ARROW-NcPt from Nano World, Matterhorn, Switzerland), with a diameter of about 20 nm, nominal resonance frequency 285 kHz, and force constant of 42 N/m were used. Measurements were performed in tapping mode at a tapping frequency of 260 kHz. Tapping amplitude was set to 80 nm (with approach at 80% of free amplitude). HNs and DNA–HNs (10 µL) samples were deposited on silicon substrate and dried under gentle nitrogen flux for 15 min. Spectra were acquired at a spectral resolution of 6 cm${}^{-1}$. A nitrogen-cooled MCT (Mercury Cadium Tellurium) detector was used to detect infrared signal. Absorption was calculated directly from Neaspec acquisition software, Neascan (see https://www.neaspec.com for details, accessed on 4 March 2021) by lock-in demodulation of the interference signal at 2nd-harmonics of the cantilevers oscillation frequency and by using as a reference Si substrate of TGQ1 sample (TipsNano, Tallinn, Estonia). Data postprocessing was performed with Neaplotter (Neaspec), and a phase correction filter was applied in order to take into account of slight phase changes of the DFG laser.

### 2.5. SEM Microscopy

Field emission scanning electron microscopy (FESEM) was carried out by a Zeiss Auriga electron microscope equipped with a Bruker Quantax detector (Energy resolution 123 eV for Mn Ka line). Twenty microliters of sample was deposited on a silicon substrate and measured after overnight evaporation. Images were acquired at 1.00–1.5 kV with secondary electrons detection, a working distance of 5 mm, and a Magnification 100–150 kX.

## 3. Results and Discussion

### 3.1. Far-Field FTIR Analysis

**Figure 2:**
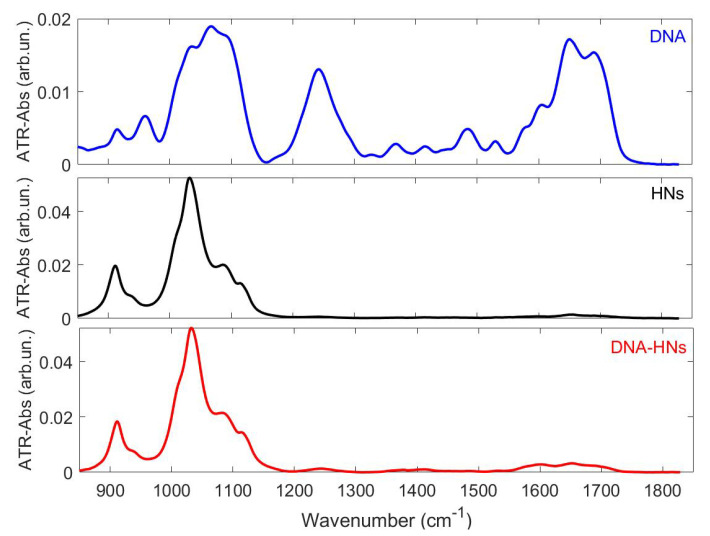
ATR-FTIR spectra of HNs (black), DNA–HNs (red) and DNA (blue) in the region between 850 and 1850 cm${}^{-1}$. For the assignment of vibrational bands, see Table 1.

In order to have an overall description of the vibrational behavior of samples to be used as reference for near-field spectra acquired with nano-FTIR spectroscopy, we measured the bulk IR features of HNs, DNA, and DNA–HNs, through the attenuated total reflection (ATR-FTIR) spectroscopy technique (see Figure 2).

The DNA spectrum shows several absorption peaks whose characteristic frequency was determined by 2nd-derivative analysis [32,33]. Strong bands are observed around 1100 cm${}^{-1}$ and 1242 cm${}^{-1}$, ascribed to phosphate stretching modes and in the region between 1660 and 1700 cm${}^{-1}$ associated with nucleobases. The absorption spectra of HNs and DNA–HNs are dominated by the absorptions of halloysite in the region around 1000 cm${}^{-1}$. Specifically, 930 cm${}^{-1}$, with a shoulder at 910 cm${}^{-1}$, was ascribed to Al${}_{2}$-OH bending, and 1032, 1121 and 1090 cm${}^{-1}$ (1088 cm${}^{-1}$ for DNA–HNs) were due, respectively, to Si-O symmetric and asymmetric stretching and Si-O-Si deformation [34]. Si-O related modes show no significant changes in DNA–HNs compared to HNs. Minor features are also found in both samples in the region between 1200 and 1700 cm${}^{-1}$, specifically at 1243 cm${}^{-1}$ (due to an overlapping of low-order Si-O moieties of halloysite and $PO_{2}^{-}$ asymmetric stretching of DNA), 1530 and 1650 cm${}^{-1}$. Additional features not observed for pristine HNs are found for DNA–HNs, whose spectral position and chemical assignments are reported in Table 1.

As can be deduced from the comparison of the absorbance extent for DNA and DNA–HNs, in the region above 1200 cm${}^{-1}$, which shows that DNA absorption is much higher than DNA–HNs, only a small fraction of the DNA mixed with HNs binds to nanotubes.

### 3.2. Nano-FTIR Analysis: DNA Phosphates and Nitrogen Bases Absorption

**Figure 3:**
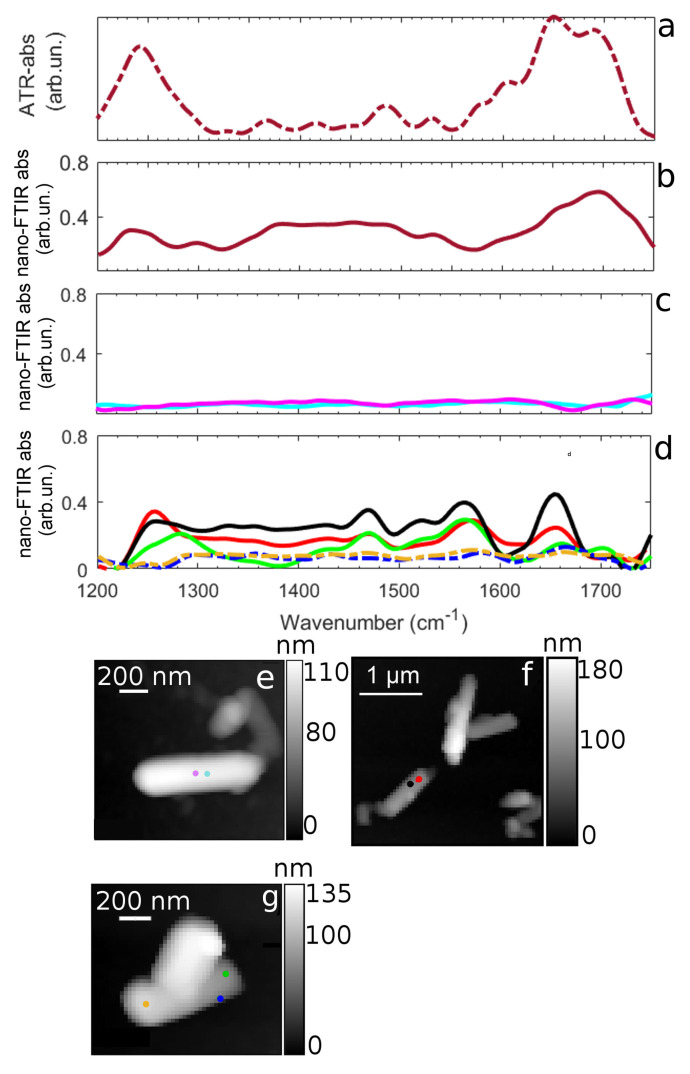
(**a**,**b**) ATR-FTIR (red dotted-line) and Nano-FTIR (red solid line) DNA absorption in the spectral region 1200–1750 cm${}^{-1}$. (**c**) Nano-FTIR absorptions at two distinct spatial points of HNs in the spectral region 1200–1750 cm${}^{-1}$. (**d**) Nano-FTIR absorptions at distinct spatial points of DNA–HNs in the spectral region 1200–1750 cm${}^{-1}$. (**e**–**g**) AFM images showing HNs (**e**) and DNA–HNs (**f**,**g**) nanotubes. Purple and light blue dots on AFM image at panel (**e**) correspond to the positions at which the spectra in panel (**c**) were acquired. Black and red dots on AFM image at panel (**f**) correspond to the positions at which the spectra of same colors reported in panel (**d**) were acquired and green, orange and blue dots on AFM image at panel (**g**) correspond to the positions at which the spectra of same colors reported in panel (**d**) were acquired.

With the aim to investigate in more detail the nature of the interaction between DNA and halloysite, providing further details about DNA structural modifications, we performed infrared measurements with nanometric spatial resolution (nano-FTIR). In order to investigate DNA adsorption on HNs’ surface and to establish the possible occurrence of DNA structural rearrangements, we focused on the spectral range between 1200 and 1750 cm${}^{-1}$, where the HN absorption is not significant. We sampled DNA, HNs and DNA–HNs by collecting the nano-FTIR spectrum at random positions. We compare in Figure 3a,b the far-field and near-field DNA spectra, respectively. The nano-FTIR spectrum resembles the spectrum observed in far-field with slight intensity variations. Therefore the, vibrational peak position of pristine DNA can be used as a reference to trace possible frequency and intensity variations in DNA–HNs near-field spectra. In Figure 3c,d nano-FTIR measurements on HNs and DNA–HNs sample are instead shown, each spectrum being measured at a specific position on the sample. HNs’ spectra (see Figure 3c) show no strong signal in this spectral range, as expected from the far-field measures in this spectral range (measured position shown in Figure 3e). Several absorption peaks are conversely found in DNA–HNs spectra (see Figure 3d with measured positions found in Figure 3f,g). As a first remark, we observe that the intensity and the shape of the DNA peaks here observed are position-dependent along the halloysite nanotube, almost disappearing in specific sample locations. This indicates a non-uniform adsorption of DNA on the HN surface. An attempt at peaks assignment is shown in Table 1. The huge peak at nearly 1242 cm${}^{-1}$ in pristine DNA and associated with the $PO_{2}^{-}$ asymmetric stretching is here blue-shifted to nearly 1253 cm${}^{-1}$. Its spectral position strongly depends on whether or not phosphate groups are in a H-bonded state [47,48]. For this reason, this band is commonly used as marker for DNA folding, hydration state and bonding state. In DNA-hydrated native state, it is typically found around 1220 cm${}^{-1}$, it shifts to around 1240 cm${}^{-1}$ when it is dehydrated [47], and higher shifts are also observed, to around 1250 cm${}^{-1}$, for cation-bound DNA [48].

Therefore, the spectral position of the DNA peak at 1242 cm${}^{-1}$ observed in pristine DNA (see Table 1) reflects the dehydrated condition at which the sample was measured (see Section 2.3 and Section 2.4 for details) [37]. The blue shift at 1253 cm${}^{-1}$ (nearly 10 cm${}^{-1}$) of this same peak in DNA–HNs, therefore, indicates an additional modification of DNA structural state induced by the interaction of DNA with HNs. This result, which has been observed on several HNs, is a first strong indication that the immobilization of DNA on the HN surface changes its conformation.

**Figure 4:**
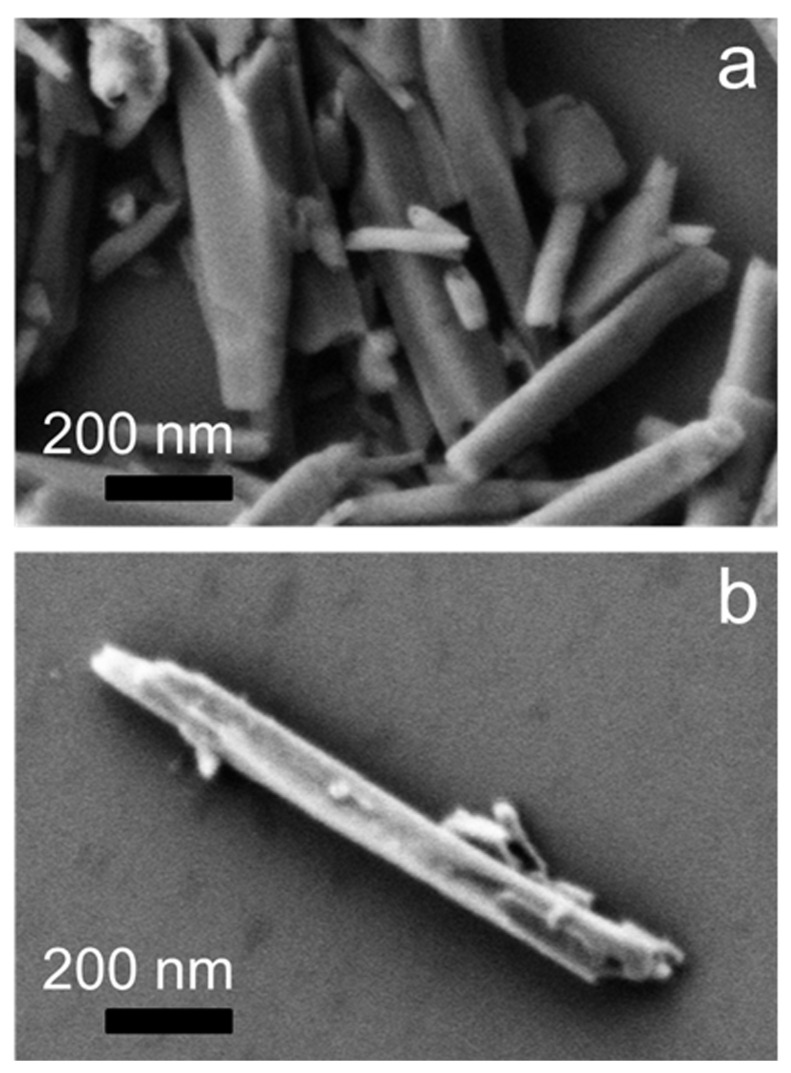
SEM images of pristine HNs (**a**) and DNA–HNs obtained after removal of DNA excess (**b**) deposited on a silicon. Bar is 200 nm. By comparison of pristine and DNA-loaded sample, the irregular appearance of the surface of DNA–HN hybrids due to the DNA layer wrapping the nanotube is immediately evident.

More importantly, we did not observe for DNA–HNs (Figure 3d) the spectral feature around 1698 cm${}^{-1}$, clearly visible in both far- and near-field spectra of pristine DNA. It is known that this band is specifically assigned to vibrations of Thymine C2=O2 moiety, and its modifications and intensity reduction are related to ligand interaction with Thymine bases present in the minor groove of DNA [43,44,49]. Previous evidence of DNA binding to amsacrine [43], an antineoplastic DNA intercalating drug, showed exactly the same behavior for the latter mode with a concomitant increase of the adenine signal around 1650 cm${}^{-1}$. This was explained by authors of [43] as the break of Thymine-Adenine base pairing through intercalation mediated by electrostatic interactions of the ligand molecule to Adenine. Moreover, we consider the peak at 1570 cm${}^{-1}$ (1575 cm${}^{-1}$ for pristine DNA), corresponding to the N-H stretching mode of Adenine. Here, we notice that this peak increases in intensity and undergoes a red shift (from 1575 to 1570 cm${}^{-1}$) when DNA is absorbed on HNs. A red shift of this mode was also observed by Elmlund et al. [41] for Adenine absorbed onto quartz surface. Therefore, the shift of $PO_{2}^{-}$ asymmetric stretching from 1242 cm${}^{-1}$ (observed in dehydrated DNA with both far-field and nano-FTIR spectroscopy) to 1253 cm${}^{-1}$ (in DNA–HNs), together with the strong intensity reduction of the 1698 cm${}^{-1}$ peak (associated to thymine C2=O2 moiety) and the intensity increase of the peak at 1570 cm${}^{-1}$ (linked to Adenine N-H) in DNA–HNs spectra, point towards the occurrence of both base-specific and backbone structural variations of DNA when adsorbed on HNs. Based on the previous results, we suggest that the break of base pairing between Thymine and Adenine occurs upon interaction; thus, positive charges (possibly on N-H groups of Adenine) can more easily be attracted by the anionic surface of nanotubes. Previous studies have suggested that H-bonds and hydrophobic interactions can all play a role in DNA adsorption on nanotubes. We suggest here that electrostatic interaction between positively charged edges of DNA and nanotubes surface constitute a further effect contributing to DNA adsorption. Since nanotubes’ surface is negatively charged, the interaction with DNA can be indeed favored by nucleotide electro-positive edges involving amino groups casting specific anion binding sites [50]. Further confirmation of the non-homogeneous DNA settlement on HNs surface comes from Scanning Electron Microscopy (SEM) images reported in Figure 4. Bare HNs (Figure 4a) show net borders and a smooth regular surface. Instead, the presence of the amorphous DNA layers gives rise to surface irregularities resembling roundish bumps and elongated lumps, which apparently detach from the nanotube borders (Figure 4b).

## 4. Conclusions

In this paper, we present the first nanoresolved infrared spectroscopic evidence of DNA immobilization on HNs. Samples of DNA–HNs were investigated through nano-FTIR, with lateral resolution of 20 nm on the nanotube surface. We deduce the presence of DNA molecules adsorbed on HNs surface by probing specific vibrational modes of DNA. In particular, the comparison between pristine DNA spectrum and DNA–HNs enlightens three main spectroscopic events occurring: (1) the peak of $PO_{2}^{-}$ vibrations at 1242 cm${}^{-1}$ shifts to 1253 cm${}^{-1}$; (2) the Thymine moiety at 1698 cm${}^{-1}$ disappears; (3) N-H mode of Adenine at 1570 cm${}^{-1}$ shifts to 1575 cm${}^{-1}$. From these observations, we conclude that DNA adsorption is favored by nucleobase interactions with nanotube surface [44,47,50] and that the structural modification at both backbone and lateral chains level also takes place upon adsorption. Thus, according to our results, DNA adsorption on HNs involves charges located in the minor groove of DNA that interact with the surface of the nanotube through electrostatic interactions. In light of a recent experiment obtained on DNA oligomers [20] and the possible chemical specificity of the DNA–HNs absorbtion we observe with the nano-FTIR technique on lateral chains (i.e., Adenine and Thymine), our results open the way for future experiments to assess the role played by specific DNA sequences in the interaction with HNs. Our research also suggests a partial covering of nanotubes by DNA molecules, and this evidence was enlightened with the unique power of nanoresolved FTIR spectroscopy, which allows us to simultaneously probe morphological nanoscale features and chemical bond vibrations.

Since relevant structural modifications in DNA adsorbed on halloysite nanocarriers are reported here, and given that deliveries from nanocarriers should guarantee optimal cargo molecules functionality, the present study highlights the constraints for the use of nanostructured clays as DNA carriers and demonstrates the power of super-resolved infrared spectroscopy as an effective and versatile tool for the evaluation of immobilization processes in the context of drug delivery and gene transfer.

## Figures and Tables

**Table 1:** Infrared peak positions and chemical group assignment for DNA, HNs and DNA–HNs obtained from the second derivative of ATR-FTIR spectra reported in Figure 2 and of DNA–HNs (nano DNA–HNs) obtained from nano-FTIR spectra reported in Figure 3.

Chemical Assignment	DNA cm${}^{-1}$	HNs cm${}^{-1}$	DNA–HNs cm${}^{-1}$	Nano DNA–HNs cm${}^{-1}$
Si-O (amorphous) [35], *PO*${}_{2}^{-}$ as [23]	1242	1243	1243	1253
C-N s (A,T) [36]	1330	-	-	1330
C-N s (C,G)) [36]	1368	-	1366	1358
Base in-plane vibration (C,G) [37,38,39]	1415	-	1415	1421
C-C C-N (G,C) [40]	1485	-	1484	1482
Base in-plane vibration (C,G)	1530	-	1530	1520
C-C C-N (C) [40], N-H (A) [41]	1575	-	-	1570
(A) [42]	1602	-	1602	-
OH (G,C,T,A)	1650	1650	1650	1652
C=O (T) [43,44]	1698	-	1698	-
C=O of nucleobases [37,39,45,46]	1707	-	1710	1706

as: asymmetric stretching; s: stretching; b: bending; A: Adenine; G: Guanine; C: Cytosine; T: Thymine.

## Data Availability

The data presented in this study are available on request from the corresponding author.
